# Postural Assessment: An Online Survey of Practicing Chiropractors in the UK

**DOI:** 10.3390/healthcare13243212

**Published:** 2025-12-08

**Authors:** Jane Johnson, Josette Bettany-Saltikov, Paul van Schaik, Julien Cordry, David Newell, Roongtip Duangkaew

**Affiliations:** 1 School of Health and Life Sciences, Teesside University, Middlesbrough TS1 3BX, UK; jane@janejohnson.co.uk (J.J.);; 2 School of Social Sciences, Humanities & Law, Teesside University, Middlesbrough TS1 3BX, UK; p.van-schaik@tees.ac.uk; 3 School of Computing, Engineering & Digital Technologies, Teesside University, Middlesbrough TS1 3BX, UK; 4 Centre of Pain and Active Inference Research, Health Sciences University, Bournemouth, Dorset BH5 2DF, UK; 5 Department of Physical Therapy, Faculty of Allied Health Sciences, Thammasat University, Bangkok 12121, Thailand

**Keywords:** postural assessment, chiropractors, practicing

## Abstract

**Background**: This study aimed to (a) determine how frequently chiropractors use postural assessment when treating patients with back or neck pain, (b) determine the rationale for the use of postural assessment by chiropractors, (c) examine the assessment methods employed, (d) explore which specific aspects of posture are assessed and (e) determine the types of back and neck conditions being treated. **Methods**: An 11-item online questionnaire was developed. A link to this survey was distributed using SurveyMonkey to the entire membership of the Royal College of Chiropractors. **Results**: There were 272 respondents to the survey. Of the respondents, 79% ‘almost always’ used postural assessment when treating patients with back or neck pain. Respondents reported using postural assessment to help determine whether a patient was making progress (61.8%), provide an outcome measure (57.1%) and help inform the diagnosis (89.2%) and treatment plan (81.8%); almost all respondents (98.1%) reported carrying out a postural assessment visually, with no aids. Most respondents reported assessing their patients posteriorly, anteriorly and from both left and right sides, observing 44 specific anatomical items. The conditions treated included sacroiliac pain (96.7%), stiff neck (95.6%), non-specific lowback pain (92.6%), lumbar disc herniation (91.5%), cervical arthritis (89.7%), lumbar strain (87.1%), lumbar arthritis (86.4%), thoracic pain (86.4%), neck strain (84.9%) and whiplash (79.8%). **Conclusions**: The findings suggest that the unaided visual assessment of posture using a large range of anatomical points is used by UK chiropractors for the purposes of aiding diagnosis and treatment of patients with back and neck pathologies.

## 1. Introduction

The assessment of posture is a common skill set within chiropractic care [1,2] and is considered to be an appropriate method of clinical examination [3]. Instructional texts intended for use by manual therapists describe the traditional method of using unaided visual assessment of posture [4,5,6,7]. Postural assessment aligns with the biomechanical model of dysfunction that has historically defined the profession [8]. It is routinely employed by physiotherapists [9,10,11,12,13] and evidence suggests similar use within chiropractic practice [14,15]. For example, in a survey of 120 physiotherapists in New York, New Jersey and Puerto Rico, 72% reported ‘frequently’ using postural correction when treating patients with neck pain [10]. Another study found that clinicians, including six chiropractors, seven physical therapists, six physiatrists, four rheumatologists and five orthopaedic surgeons, assessed spinal posture ‘regularly’ [11]. Likewise, a focus group of Portuguese physiotherapists reported ‘routinely’ assessing head posture in patients with neck pain [13,14]. Comparable findings have been reported among chiropractors: in Belgium, 72 of 80 respondents reported ‘often to always’ using visual posture analysis [14,15], while in Saskatchewan, 79% of 62 respondents ‘always’ documented visual observation of posture [15].

Poor posture is recognized as a risk factor for musculoskeletal disorders and pain [16], and in athletes it may impair movement efficiency [17]. However, a limitation of many studies [10,11,14,15] is the lack of quantification of terms such as ‘often’, ‘regularly’ and ‘routinely’. A Canadian survey addressed this by quantifying frequency: of 500 respondents, 34.4% reported using postural assessment ‘always’ (76–100% of the time), 28.2% ‘usually’ (51–75%) and 18.6% ‘often’ used it (26–50%) [18]. In the UK, a survey of 2448 chiropractors found that 97.1% considered it reasonable to discuss posture improvement with patients, 96.3% gave advice about poor posture and 80.4% set goals and re-evaluated progress [19].

Small-scale studies indicate that postural assessment by physiotherapists and chiropractors is predominantly visual and unaided. For example, Fedorak et al. (2003) reported that 93% of respondents used visual assessment [11]; Silva et al. (2009) found that 100% of Portuguese physiotherapists relied on observation [12], and Van Schaik et al. (2002) reported that 84% of physiotherapists in Teesside used unaided visual assessment [20]. Similarly, Hinton’s survey found that 79% of chiropractors used visual assessment [15], while Ailliet et al. (2010) reported 72% of therapists did so [14]. More recently, Martin et al. (2024) surveyed physiotherapists, osteopaths and chiropractors in the UK, finding that visual assessment remains the predominant method [3].

Posture may be assessed from the front, back or side views, with specific attention to the relationships between body segments. While little is known about the precise methods used by chiropractors, it is reasonable to assume that they employ techniques taught during undergraduate training. Harrison et al. (2000) state that all chiropractic, osteopathic, physical therapy and medical colleges teach a plumb line analysis [21]. At AECC University College, training documents describe the traditional method of visually observing posture from multiple views and recording findings on a body chart [22]. These documents also employ subjective terminology familiar to manual therapists (e.g., ‘round shoulders’, ‘elevated shoulders’, ‘forward-drawn head’). Despite concerns about the validity [23] and reliability [11] of unaided visual assessment, chiropractors appear to continue using this method, consistent with traditions in physiotherapy and osteopathy.

In summary, the frequency, rationale and methods of postural assessment used by UK chiropractors remain largely unknown. Greater understanding in this area could inform a more evidence-based approach to practice. To the authors’ knowledge, this is the first survey to examine in detail the frequency and specific methods of postural assessment among UK chiropractors. The purpose of the study was to evaluate how often chiropractors perform postural assessment, the rationales underpinning its use and the specific methods employed, including the extent of standardization across the profession. By addressing these questions, the study aims to provide new insights into the role of postural assessment in contemporary chiropractic care and to contribute to the development of evidence-based practice.

## 2. Materials and Methods

### 2.1. Participants

The entire membership of the Royal College of Chiropractors (RCC) was invited to take part (n = 1206). The rationale for selecting RCC members was that they have a large number of members who could easily be accessed.

### 2.2. Measures and Instrumentation

#### 2.2.1. Theoretical Framework

The development of the survey was guided by the traditional method of postural assessment taught in chiropractic education, which emphasizes unaided visual observation of posture from the front, back and side views, with documentation using a body chart [21,22]. This approach aligns with the biomechanical model of dysfunction that has historically defined chiropractic practice [8] and is widely reported as a routine component of clinical examination among physiotherapists and chiropractors [9,10,11,12,13,14,15]. Poor posture is recognized as a risk factor for musculoskeletal disorders and pain [16], further supporting its clinical relevance. Thus, the survey was designed to reflect the clinical decision model in which postural assessment is used to identify dysfunction, guide treatment planning and provide advice on posture improvement.

#### 2.2.2. Questionnaire Development and Pre-Testing

A new 11-question multiple-choice, cross-sectional online survey was developed. Following a pilot study conducted with clinicians and the supervisory team (n = 10), minor revisions were made. Specifically, the answer options for Question One were quantified using percentages consistent with those employed by Puhl et al. (2015) [18]. The options were defined as follows: ‘almost always’ (76–100% of the time), ‘frequently’ (51–75%), ‘some of the time’ (26–50%) and ‘rarely’ (1–25%). The inclusion of percentages served two purposes: first, to clarify the meaning of these frequency terms and facilitate accurate responses from chiropractors; second, to enable direct comparison with the findings of Puhl et al. (2015) [18].

The web survey design incorporated short, fixed pages rather than scrolling pages, and all questions were mandatory except those deemed irrelevant to a given participant. A skip function was implemented, as recommended by Peytchev et al. (2006) [24]. For example, if a participant indicated that they never used postural assessment, subsequent questions relating to its use were automatically skipped. The participant was then directed to the final two questions, which asked whether they wished to provide additional comments on postural assessment and to identify their profession.

The rationale for these design choices was twofold. First, Peytchev et al. (2006) demonstrated that fixed pages are quicker to complete than scrolling formats [24]. Second, automated skip logic reduces errors of omission and commission. An error of commission refers to performing an incorrect action [25]—for instance, mistakenly selecting that posture is observed from the front view when it is not. An error of omission refers to failing to perform an action that should have been completed [25]—for example, neglecting to select that posture is observed from the front view when it is.

#### 2.2.3. Questionnaire

Postural assessment items listed in the survey were constructed using the limited literature available, the AECC University College training document [22], two texts on postural assessment aimed at manual therapists [4] and the clinical experience of the author. This survey focused on the chiropractic assessment of a patient during their first appointment, as opposed to assessment as part of ongoing treatment. Questions asked about the frequency (objective 1) and rationale (objective 2) for the use of postural assessment, methods of use (objective 3), which specific aspects of posture were assessed (objective 4) and the sorts of pathology chiropractors routinely came across in practice (objective 5). One question was open-ended and asked whether the participant wished to add any further comments. The final question asked the participant to select the professional category (chiropractor, osteopath, physiotherapist, sports therapist, other) with which they identified, as the survey was later intended for use with other manual therapists. Questions 2, 3, 4, 6, 7, 8, 9 and 10 of the survey contained free-text boxes, but there were no free-text options for questions 1, 5 and 11 (Table 1).

#### 2.2.4. Data Collection Procedure

A participant’s information sheet was created and ethical approval sought from the AECC University College in February 2016. The AECC University College responded that the survey fell beneath their criteria for risk, and ethical approval was granted. During April and May 2016, a link to the survey was disseminated via SurveyMonkey to 1206 members of the Royal College of Chiropractors (1151 based in the UK and 55 based overseas).

### 2.3. Data Analysis

For questions that generated numerical data, descriptive analysis of responses to each answer was converted to percentages. In particular, descriptive analysis was performed for questions relating to the frequency of use of postural assessment, rationale for use of postural assessment, method used to assess posture, specific postural assessment indices observed and back and neck pathologies encountered.

For free-text questions, content analysis was used: a coding framework was developed to determine whether any themes emerged from written responses. In order to ensure consistency of coding by raters, a Manual for Raters was created. This manual consisted of a set of coding rules as advocated by Zhang and Wildemuth (2009) [26]. Repetition of words or phrases is one of the easiest methods to identify themes [27] and this method was used to develop themes. The author and another manual therapist cut and sorted the free-text, identifying important expressions, and arranged these in thematic piles. For each question, emergent themes were each assigned an alphabetic code. In the example of question 2, there were three themes labelled A, B and C. Coding of free-text responses was then performed by two independent reviewers. That is, each reviewer assigned responses to questions to one or more of the codes. Discrepancies were resolved by a third reviewer.

## 3. Results

### 3.1. Quantitative Analysis

**Figure 1:**
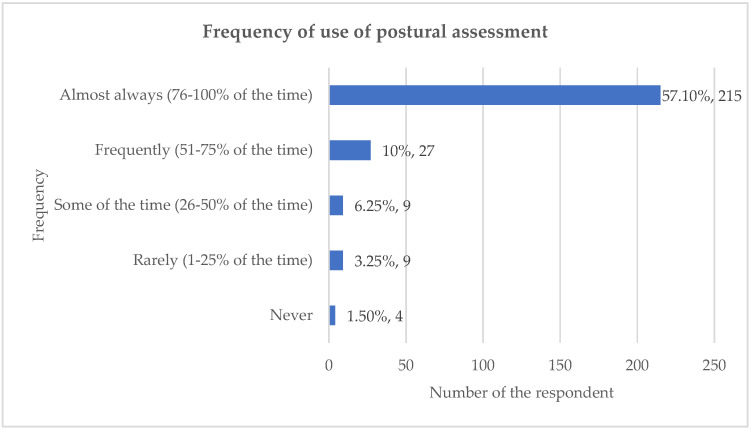
Frequency of use of postural assessment.

There were 272 respondents to the survey, yielding a response rate of 22%. The frequency data indicated that postural assessment is a central component of clinical practice for UK chiropractors: 79% of respondents reported using postural assessment ‘almost always’ (76–100% of the time) when treating new patients with back or neck pain (Figure 1).

#### 3.1.1. Rationale for the Use of Postural Assessment: Informing Clinical and Patient Management

The rationale for using postural assessment generally fell into two primary areas: informing the clinician’s decision-making and informing the patient’s understanding.

##### Informing Clinical Decisions

**Figure 2:**
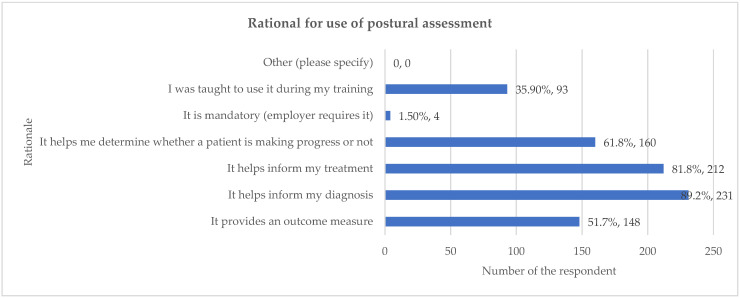
Rationale for the use of postural assessment. Percentages may exceed 100% because respondents were able to select more than one option for these multiple-choice questions.

The majority of respondents indicated that postural assessment was used to inform core clinical decisions. Specifically, 89.2% reported that it helped inform their diagnosis, and 81.8% stated it helped inform the treatment plan. Additionally, over half of the respondents used the assessment for ongoing management: 61.8% used it to determine whether a patient was making progress, and 57.1% reported that it provided an outcome measure (Figure 2). Percentages may exceed 100% because respondents were able to select more than one option for these multiple-choice questions.

##### Informing Patient Management

Qualitative analysis revealed that respondents also utilized postural assessment as a tool for patient education and adherence. One theme that emerged was that the assessment was used because it informs the patient (e.g., “It helps to educate the patient”) or because patients find it important (“Patients understand it and think it is important”). Other rationales included using the assessment because it alerts the clinician to “poor postural habits that need to be addressed and advised against”.

#### 3.1.2. Methods of Assessment and Anatomical Items Observed

**Figure 3:**
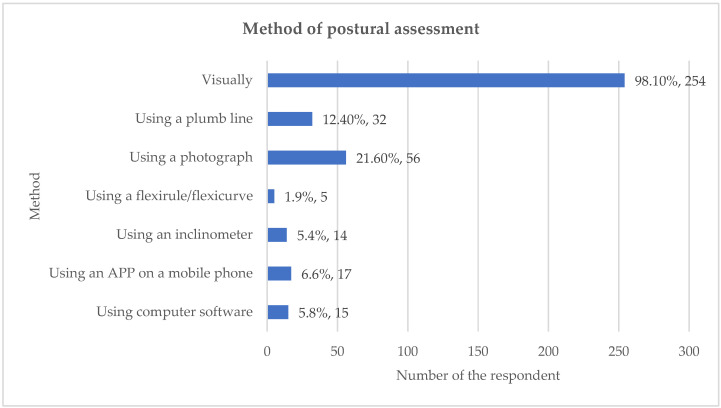
Method of postural assessment. Percentages may exceed 100% because respondents were able to select more than one option for these multiple-choice questions.

**Figure 4:**
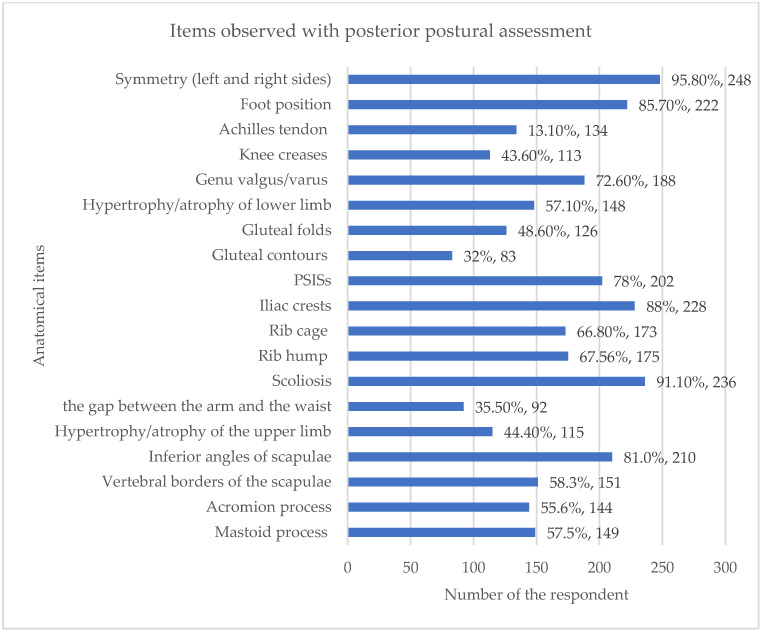
Items observed with posterior postural assessment. Percentages may exceed 100% because respondents were able to select more than one option for these multiple-choice questions.

The primary method employed by the 259 respondents who used postural assessment was unaided visual observation, reported by 98.1% of chiropractors. This reliance on visual assessment was significantly higher than the use of measurement devices such as a photograph (21.60%) or a plumb line (12.40%). Respondents routinely assessed posture from multiple cardinal views. The most frequent views used were posterior view (96.1%; n = 249), anterior view (79.9%; n = 207) and both left and right lateral sides (71.8%; n = 186) (Figure 3). A wide range of anatomical indices were observed. When assessing posture posteriorly, the most frequently observed item was symmetry between left and right sides of the body (95.8%). During lateral assessment, items related to the spine and head were nearly universally observed, including head position (98.1%), shape of the thoracic spine (91.5%) and shape of the lumbar spine (90.7%) (Figure 4). Percentages may exceed 100% because respondents were able to select more than one option for these multiple-choice questions.

#### 3.1.3. Pathologies Treated and Potential Relationship to Assessment Focus

**Figure 5:**
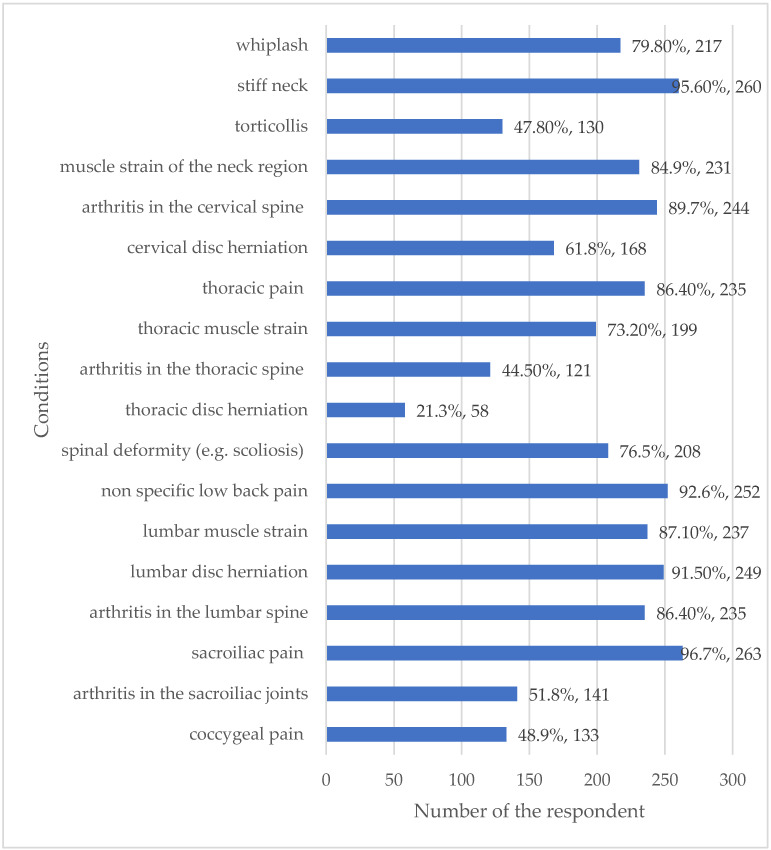
Back and neck pathologies commonly encountered. Percentages may exceed 100% because respondents were able to select more than one option for these multiple-choice questions.

The pathologies commonly treated by the respondents demonstrate a clinical focus on both soft tissue and degenerative spinal conditions. The most frequently reported conditions included sacroiliac pain (96.7%), stiff neck (95.6%), non-specific low-back pain (92.6%) and lumbar disc herniation (91.5%) (Figure 5).

##### Comparison Between Variables (Assessment Indices vs. Pathologies)

The high frequency of treating pathologies that often involve spinal structure and symmetry (e.g., sacroiliac pain at 96.7% and lumbar disc herniation at 91.5%) corresponds directly with the highest frequency of assessment indices observed, such as symmetry between left and right sides (95.8%) and the position of the pelvis (85.3%). Furthermore, the observation of head position (98.1%) and shape of the cervical spine (90.0%) aligns with the high prevalence of stiff neck (95.6%) and cervical arthritis (89.7%) being treated. This suggests that assessment parameters are tailored to the most common spinal pathologies encountered in UK chiropractic practice.

### 3.2. Qualitative Analysis

Eight survey questions (questions 2–4, 6–10) contained free-text options. Of these, seven received free-text responses. Table 2 shows how many responses were obtained for each of the free-text questions and the percentage level of agreement between raters for the assignment of codes for each response. There were 229 free-text responses. The coding process was used to explore emerging themes from these responses. The codes were assigned by independent raters using the Manual for Raters, and the percentage of agreement be-tween raters for each response was calculated (Supplementary Materials).

#### 3.2.1. Rationale for the Use for Postural Assessment

Question 2 was

As you answered ‘always’, ‘frequently’ or ‘some of the time’ to the previous question, please tick as many of the statements as you agree with. I use postural assessment because:

Responses to Question 2 fell predominantly into two themes: Postural assessment was used because, first, it informs the clinician or was for their benefit.

*“It alerts me to poor postural habits that need to be addressed and advised against”*

[Participant 132];

*“It helps inform my rehabilitation protocols”*

[Participant 25].

Second, it informs the patient or is for the benefit of the patient.

*“Patients understand it and think it is important”*

[Participant 148];

*“It helps to educate the patient”*

[Participant 74].

Question 3 was

As you answered ‘rarely’ or never’ to the previous question, please tick any of the following statements you agree with. I rarely or never carry out postural assessment because: I’m wondering if this was because it may have been seen as being repetitive?

#### 3.2.2. Methods Used to Carry out Postural Assessment

Question 4 was

What methods do you use to carry out postural assessment?

Eight methods were identified from responses to this question, and each method was used as a theme to code the responses. The eight methods were an ‘app’ (n = 2), a ‘goniometer’ (n = 1), ‘a grid or chart’ (n = 1), ‘mirror’ (n = 2), ‘palpation’ (n = 2), ‘photograph’ (n = 1), ‘Spinal Analysis Machine’ (n = 4) and ‘x-ray’ (n = 3). One respondent commented that they did not use any technology to assess posture.

#### 3.2.3. Positions Used for Postural Assessment

Question 6 was

If you perform POSTERIOR postural assessment with back and neck pain patients, what are the things you observe?

Postural indices reported were wide-ranging and included those relating to the head (n = 11) and the relationship between one body part and another (n = 11). For example, ‘ear level’ and ‘lordosis/kyphosis relationship’, respectively.

Question 7 was

If you perform ANTERIOR postural assessment with back and neck pain patients, what are the things you observe?

The postural indices reported were again wide-ranging and included indices relating to the lower limb (n = 6) such as ‘foot arch’ and ‘hip rotation’ and body shape or size (n = 4) such as ‘abdomen size’ and ‘abdominal contours’.

Question 8 was

If you perform LATERAL postural assessment with back and neck pain patients, what are the things you observe?

Responses to this question included those relating to the position of the body and weight distribution (n = 6). For example, ‘balance of anterior/posterior weight’ and ‘whether the weight is being carried forward onto the toes’. All of the themes for responses to Questions 6, 7 and 8 are listed in the Manual for Raters.

#### 3.2.4. Back and Neck Pathologies Encountered in Clinical Practice

Question 9 was

Thinking only about patients with back or neck pain, what sorts of pathologies do you come across commonly in your practice?

In response to Question 9, in addition to the pathologies listed, the participants stated other pathologies in the free-text box available for this purpose, including conditions affecting the face, head and neck (dizziness, headaches, sinusitis), upper limb (brachial plexus problems, thoracic outlet syndrome), chest/ribs (costotransverse joint sprain), spine (dural tube problems, spinal stenosis), pelvis (perineal pain), lower limb (piriformis syndrome, sciatica) and conditions not specific to a part of the spine (cancer, neuralgia, tension).

#### 3.2.5. Additional Comments

Question 10 was,

Is there anything else you would like to say about your use of postural assessment?

Responses to question 10 were wide-ranging and fell within four broad themes. The first theme was regarding the methods of postural assessment: below are a few of the statements that participants mentioned.

*“How can you assess posture without xray? You can look visually at someone and their structure on xray can be very different to what you expect. You must understand spinal coupling mechanisms and 3D analysis if you wish to correct posture photographs like x-rays can project distortion especially if they are not taken correctly”*

[Participant 74];

*“I combine it with dynamic assessment and gait”*

[Participant 238].

The second theme included comments made regarding specific aspects of posture that were reported to be observed during the assessment:

*“I also look for torque, *i.e.,* rotation in the horizontal plane, of the head, shoulder and pelvic girdles in relation to each other and to the position of the feet”*

[Participant 73].

The third theme comprised comments that were made regarding the use and value of postural assessment (both positive and negative).

*“It is an absolute must in practice for us and the patient.”*

[Participant 55];

*“I think it is way overrated.”*

[Participant 253]

Finally, the fourth theme comprised comments about the rationale for postural assessment:

*“Posture is adapted, neutral and, unique to the individual—to not assess posture is doing an unforgivable disservice to the patient and not fulfilling our remit of neuro-musculoskeletal consultants.”*

[Participant 257]

## 4. Discussion

### 4.1. Key Findings

The results of this study demonstrated that the majority of respondents reported carrying out postural assessment almost all of the time on patients with neck and back conditions using visual assessment alone. This was conducted for the purposes of informing their diagnosis and treatment of patients with back and neck pain. Over 50% of participants reported that they used it to provide a clinical outcome measure. Generally, chiropractors in this survey reported observing their patients from the posterior, anterior and lateral views in order to carry out the assessment, observing a large number of different indices. The most common indices of these were symmetry, spine shape and the position of the head and pelvis, as well as the position of the shoulders, knees and feet. The back and neck presentations most commonly encountered were sacroiliac pain, stiff neck, non-specific low-back pain, herniation of a vertebral disc in the lumbar spine, arthritis of the cervical and lumbar spine, muscle strain of the lumbar spine or neck, thoracic pain and whiplash. It is not known whether the response rate of 22% for this survey falls within the norm for surveys of chiropractors.

The response rate of this study is similar to that of Fikar et al. (2015) (22%) [19] but lower than those of Puhl et al. (2015) (68%) [18] and Alliet et al. (2010) [14]. It seems that postal questionnaires—of which the latter two citations are examples—might generate higher response rates. Secondary analysis by Russell et al. (2004) of data from 46 published postal surveys of chiropractors ranged from 7% to 91.4% (mean 52.7%) [28].

### 4.2. Quantitative and Qualitative Data

This study reported that 79% (n = 215) of respondents ‘almost always’ (76–100% of the time) used postural assessment when treating patients with back or neck pain. The only study found to quantify the frequency for the use of postural assessment by chiropractors is that by Puhl et al. (2015) [18]. That study details that 29.9% (n = 146) of respondents ‘always’ (76–100% of the time) used postural assessment and 20.7% (n = 101) ‘usually’ (51–75%) used it. That is, 50.6% (n = 247) of chiropractors in the Puhl et al. (2015) study [18] usually always (75–100%) used postural assessment, compared to the researcher’s study, which found 79% of respondents almost always used it. The reason for the discrepancy between the frequency of use of postural assessment reported in the two studies is unknown. One reason may be any training variations between chiropractors working in different geographical regions: the researcher’s study surveyed UK chiropractors and Puhl et al. (2015) surveyed Canadian chiropractors [18].

In the study by Alliet et al. (2010), 72% (n = 80) of Belgian chiropractors reported ‘often to always’ using postural assessment, and 97.1% of UK chiropractor respondents reported evaluating posture [14]; however, in both cases, the description of frequency was not quantified, making comparisons difficult. In another study, 79% (n = 48) reported ‘always’ using visual assessment of posture with a new patient [15]. Similarly, three studies in which the use of postural assessment by clinicians was embedded within the questions put to participants failed to quantify the frequency of use. Enwemeka et al. (1986) surveyed 120 physiotherapists in New York, New Jersey and Puerto Rico and reported that 72% (n = 33) of respondents stated that they ‘often’ used postural correction when treating patients with neck pain [10]. A focus group study carried out in Portugal involving 21 physiotherapists used a variety of set questions, one of which was, ‘Do you assess head posture for patients with neck pain in your clinical practice?’ [12]. The authors stated that physiotherapists reported routinely assessing head posture for patients with neck pain. In the study of a mixed group of twenty-eight clinicians (six chiropractors, seven physical therapists, six physiatrists, four rheumatologists and five orthopaedic surgeons), Fedorak et al. (2003) detailed that all participants reported regularly using visual spinal posture assessment, with 93% saying this was the tool they used most frequently to assess spinal posture [11].

This study found that almost all respondents (98.1%) relied on unaided visual assessment when performing postural evaluations. This finding is consistent with small-scale studies involving chiropractors and physiotherapists in other regions, which reported high rates of visual assessment, such as 84% in Teesside [20], 93% in Canada [11] and 100% in Portugal [12]. The General Chiropractic Council also indicates that chiropractors are taught to assess posture visually without measurement devices. However, the high reliance on unaided visual assessment must be interpreted cautiously, as this methodology is known to lack validity and reliability when assessing spinal posture. While the visual assessment method may be a tradition amongst physiotherapy and osteopathy, the continuation of this practice raises questions, particularly since the evidence supporting a clear relationship between static posture and pain is limited. It is possible that this tradition persists because, until recently, practical alternatives to visual assessment have been unavailable.

This study identifies the rationale for the use of postural assessment by chiropractors, with almost 90% of respondents reporting that they used it to inform their diagnosis. Over 80% said that it helped inform their treatment and over 50% stated that it provided an outcome measure. This is consistent with the findings of Alliet et al. (2010), who reported that 72% (n = 80) of Belgian chiropractors used postural analysis as a diagnostic procedure [14]. Of English-speaking Canadian chiropractors, 71.5% (n = 339) of used it for this reason [18], with 80.4% (n = 409) of UK chiropractors setting goals and re-evaluating progress with regard to patient posture [19]. Additionally, Puhl et al. (2015) reported that over 80% (n = 406) used ‘posture’ as a treatment method [18]. This may be in the form of exercises to correct posture.

The strong quantitative data showing that postural assessment is used to inform diagnosis (89.2%) and treatment (81.8%) is consistent with findings from Canadian and Belgian chiropractors. Crucially, the qualitative findings illuminate why this diagnostic role is so highly valued: the assessment serves a dual purpose. Beyond informing the clinical decision-making process (e.g., alerting the clinician to poor postural habits or informing rehabilitation protocols) [29,30], it is also used as a critical communication tool for patient education and buy-in. Participants noted that postural assessment “helps to educate the patient” and is used because “patients understand it and think it is important”. This suggests that the value of the assessment may be perceived less in terms of structural biomechanical accuracy and more in terms of clinical utility and patient engagement, despite recognized limitations in its validity.

This study also identified specific anatomical points that were routinely observed by chiropractors carrying out postural assessments. When assessing patients with back or neck pain, the majority of respondents reported observing symmetry between the left and right sides of the body, head position, shape of the spine, iliac crests and position of the pelvis. They also assessed for the presence of scoliosis, as well as foot position. Over 70% reported looking at the position of the shoulder and scapula and whether there was evidence of genu valgum or genu varum. No studies were found with which to contrast these findings.

This survey of UK chiropractors revealed specific back and neck pathologies that UK chiropractors were treating, with over 90% treating patients with sacroiliac pain, stiff neck, lumbar disc herniations and non-specific lower-back pain.; over 85% of chiropractors treated arthritis in the cervical or lumbar spines, thoracic pain and muscle strain in the lumbar region. A similar study by Ailliet et al. (2010) reported that 85.5% of Belgian chiropractors treated patients whose primary complaint was lower-back and/or neck pain [14].

The finding that respondents observe a large number of specific anatomical items across posterior (96.1%), anterior (79.9%) and lateral views is unique, as previous studies lack such specificity. This wide-ranging observation (e.g., 44 specific anatomical items) supports the general conclusion that UK chiropractors employ a traditional, comprehensive, multi-view approach to static posture assessment. The fact that observations focus heavily on symmetry, head position and spine shape confirms a strong biomechanical focus, which aligns with the fact that they treat a high volume of related spinal conditions, such as sacroiliac pain (96.7%) and stiff neck (95.6%).

While visual postural assessment remains a common skill within chiropractic practice, its reliability and validity are limited. Contemporary evidence increasingly emphasizes that static posture alone has poor predictive value for musculoskeletal pain outcomes. Current biopsychosocial perspectives highlight that pain is influenced by a complex interaction of biological, psychological and social factors and cannot be explained solely by structural or postural deviations. For example, recent studies suggest that although poor posture may contribute to discomfort or inefficiency in movement, it is not consistently associated with the onset or persistence of pain.

Accordingly, the findings of this survey should be interpreted within this broader evidence context. The high reliance on unaided visual assessment underscores the need for chiropractic education and continuing professional development to integrate evidence-based approaches that reflect biopsychosocial models of care. This would help practitioners move beyond purely structural interpretations of posture and toward a more holistic understanding of patient outcomes.

### 4.3. Limitations of the Study

The survey achieved a 22% response rate, which is considered low for survey research and increases the likelihood of non-response bias. However, our response rate was 22%, which is in the range of online surveys generally [31]. Because no information was available regarding the characteristics of non-respondents, the representativeness of the sample cannot be determined. It is possible that respondents were those with a greater interest in posture, which may have influenced the findings. This limitation should be considered when interpreting the results. In particular, enthusiastic participation among clinicians who value posture assessment may have inflated reported usage rates. Consequently, the actual use of postural assessment may be less common than our results suggest. This limitation also applies to other studies [19] reporting similar response rates. In our study, neither stratified nor random sampling methods were employed. It was not possible to perform stratified sampling. This is because we did not have access to the members’ demographics that would be needed to perform stratification. Moreover, given the size of the member population, it would not have been feasible to collect and analyse the data even if the response rate were 100% (N = 1206). Therefore, there was no valid reason to perform stratified sampling from the member population. Random sampling was not suitable because this would have further reduced the sample size.

Several factors may explain the low response rate in this study. First, no advance warning of the survey was provided, an approach believed to increase participation [32]. Second, practitioners may receive large volumes of email, some of which are ignored. A high rate of non-responses also increases the probability of statistical bias [33]. However, Cook et al. (2000) argue that in survey research, response representativeness is more important than response rate [34]. The implication is that the study may need to be repeated to confirm the findings.

Unanswered questions remain. It is not clear why the majority of respondents reported using visual postural assessment when this method is known to lack objectivity and may therefore be neither valid nor reliable. This may reflect its historical role in assessment processes and the limited availability of practical alternatives until recently. Similarly, it is unclear why a large proportion of respondents reported using postural assessment to inform diagnosis and treatment of back or neck pain, given the limited evidence supporting a relationship between posture and pain.

Although the survey was piloted with 10 participants, the pilot was primarily used to refine wording and structure rather than to establish psychometric properties. No formal validity or reliability analyses (e.g., content validity, test–retest reliability or internal consistency such as Cronbach’s alpha) were conducted. This represents a methodological limitation, as the absence of validity and reliability testing restricts confidence in the robustness of the findings. Future research should therefore incorporate systematic validation procedures to strengthen the credibility and generalizability of survey results.

Further investigation is warranted. Interviews and focus groups could provide deeper insights into clinicians’ rationales for using postural assessment. Comparative studies are also needed to determine whether these findings correspond with methodologies employed in related manual therapy professions, such as physiotherapy and osteopathy. Moreover, additional demographic variables (e.g., years in practice, type of clinical setting) were not collected in this survey. As a result, the findings cannot be contextualized against these factors, which may limit interpretation of practice patterns. Future studies should incorporate these variables to provide a more comprehensive understanding of chiropractic practice.

While our findings highlight ongoing concerns regarding the reliability and validity of postural assessment, they also point to several directions for future work. Research should prioritize the use of standardized and validated assessment tools to enhance consistency across studies and clinical settings. Longitudinal designs are needed to establish the predictive validity of postural measures in relation to musculoskeletal outcomes. Incorporating objective technologies such as motion capture, wearable sensors or three-dimensional imaging may further improve measurement accuracy. In addition, studies examining inter-rater reliability across diverse populations would strengthen the generalizability of postural assessment methods.

Finally, although respondents mentioned the use of apps, photography and other digital tools, the survey did not capture detailed quantitative data on the frequency or intensity of their use. As a result, the findings may not fully reflect the extent of technological integration in clinical practice. Another limitation is that some respondents appeared to conflate static postural assessment with dynamic functional assessment, which may have introduced interpretive overlap in the qualitative data.

## 5. Conclusions

The findings of this study clearly indicate that postural assessment is a well-established clinical practice among UK chiropractors, primarily used to support diagnosis and inform treatment planning. The predominant method employed is unaided visual observation.

Practitioners consistently reported conducting evaluations from multiple cardinal planes—posterior, anterior and lateral—while observing a wide range of specific anatomical landmarks. The information gathered through this process is used both to guide therapeutic decisions and to educate patients about their musculoskeletal health. Additionally, the survey data revealed that these practitioners manage a broad and complex spectrum of back and neck pathologies.

These results from the cross-sectional study highlight the need for further scholarly investigation into the clinical rationale and decision-making frameworks that underpin the routine use of postural assessment. Comparative research is also warranted to explore whether these findings align with practices in other manual therapy disciplines, such as physiotherapy and osteopathy. One possible inference is that the reliance on visual assessment stems from a perceived lack of validated, logistically feasible alternatives for dynamic, real-time use in typical clinical settings. Given chiropractors’ reliance on unaided visual assessment, chiropractic education should reinforce training in observational diagnostics, as well as in objective photographic techniques. This includes surface photography methods such as Final Surface and artificial intelligence–based posture assessment. Continuing professional development should support the refinement of these skills and promote the integration of evidence-based methods to enhance clinical accuracy and practitioner confidence.

## Figures and Tables

**Table 1:** The 11 questions asked in the questionnaire.

Question
Q 1. Please tick ONE box to indicate how frequently you use postural assessment with a NEW patient who comes to you with back or neck pain.
Almost always (76–100% of the time)
Frequently (51–75% of the time)
Some of the time (26–50% of the time)
Rarely (1–25% of the time)
Never
Q2. As you answered ‘always’, ‘frequently’ or ‘some of the time’ to the previous question, please tick as many of the statements you agree with. I use postural assessment because:
It provides an outcome measure
It helps inform my diagnosis
It helps inform my treatment
It helps me determine whether a patient is making progress or not
It is mandatory (employer requires it)
I was taught to use it during my training
Other (please specify)…………………………………………….
Q3. As you answered ‘rarely or ‘never’ to the previous question, please tick any of the following statements you agree with. I rarely or never carry out postural assessments because:
I don’t have time
Postural assessment is not relevant to my diagnosis or treatment
I don’t believe it is accurate or objective
My patients don’t like it or it is not appropriate for them
I was not taught to use it
Other (please specify)…………………………………………….
Q4. What methods do you use to carry out postural assessment? You may choose more than one option or tick as many as apply.
Visually
Using a plumb line
Using a photograph
Using a flexirule/flexicurve
Using an inclinometer
Using an APP on a mobile phone
Using computer software
Other (please specify)…………………………………………….
Q5. From which positions do you assess your subject?You may choose more than one option.
Both left and right sides
Right lateral only
Left lateral only
Posterior
Anterior–Posterior
Q6. If you perform POSTERIOR postural assessment with back and neck pain patients, what are the things you observe? You may choose more than one option.
Symmetry between left and right sides of the body
Foot position
Achilles tendon
Knee creases
Genu valgus/varus
Hypertrophy/atrophy of lower limb muscles
Gluteal folds
Gluteal contours
PSISs
Iliac crests
Rib cage
Rib hump
Scoliosis
Size of the keyhole (gap between the upper limb and thorax)
Hypertrophy/atrophy of the upper limb
Inferior angles of scapulae
Vertebral borders of the scapulae (distance laterally from the spine)
Acromion process
Mastoid process
Other (please specify) …………………………………………….
Q7. If you perform ANTERIOR postural assessment with back and neck pain patients, what are the things you observe? You may choose more than one option.
General symmetry between left and right sides of the body
Feet
Knees
Genu valgus/varus
Hypertrophy/atrophy of lower limb muscles
Greater trochanters
ASISs
Iliac crests
Rib cage
Size of the “keyhole” (gap between the upper limb and thorax)
Hypertrophy/atrophy of upper limb muscles
Clavicles
Acromioclavicular joints
Muscles of the neck
Mastoid processes
Facial symmetry
Other (please specify) …………………………………………….
Q8. If you perform a LATERAL postural assessment with back and neck pain patients, what are the things you observe? You may choose more than one option.
Head position
Shape of cervical spine
Shape of thoracic spine
Shape of lumbar spine
Position of pelvis—anterior or posterior pelvic tilt
Shoulder position
Position of upper limb
Position of lower limb—e.g., genu recuravtum/genum flexum
Overall body shape
Other (please specify) …………………………………………….
Q9. Thinking only about patients with back or neck pain, which sorts of pathologies do you come across commonly in your practice? Please note, the following are only some of the possible pathologies you may commonly come across. (Please tick as many as apply and use the ‘other’ box to add others)
whiplash
stiff neck
torticollis
muscle strain of the neck region
arthritis in the cervical spine
herniation of an intervertebral disc in cervical spine
thoracic pain
muscle strain of the thoracic region
arthritis in the thoracic spine
herniation of an intervertebral disc in thoracic spine
spinal deformity (e.g., scoliosis)
non-specific low back pain
muscle strain of the lumbar spine
herniation of an intervertebral disc in lumbar spine
arthritis in the lumbar spine
sacroiliac pain
arthritis in the sacroiliac joints
coccygeal pain
Other (please specify) …………………………………………….
Q10. Is there anything else you would like to say about your use of postural assessment?
(please specify) …………………………………………….
Q11. Finally, please select the answer that most closely represents your profession:
Chiropractor Osteopath Physiotherapist
Sports Therapist
Osteopath
Physiotherapist
Other (please specify) …………………………………………….

**Table 2:** Number of responses to questions with a free-text option.

Survey Question	Number of Free-Text Responses	Percentage (%) Agreement
1. No free-text option	NA	NA
2. As you answered ‘always’, ‘frequently’ or ‘some of the time’ to the previous question, please tick as many of the statements you agree with. I use postural assessment because: R where are the statements they were responding to?	28	21 had 100% agreement 2 had 50% agreement 5 had no agreement
3. As you answered ‘rarely’ or ‘never’ to the previous question, please tick any of the following statements you agree with. I rarely or never carry out postural assessment because:	0	NA
4. What methods do you use to carry out postural assessment? You may choose more than one option or tick as many as apply.	17	16 had 100% agreement 1 had no agreement
5. No free-text option	NA	NA
6. If you perform POSTERIOR postural assessment with back and neck pain patients, what are the things you observe? You may choose more than one option.	31	24 had 100% agreement 1 had 40% agreement 1 had 33% agreement 2 had 25% agreement 1 had 20% agreement 2 had zero agreement
7. If you perform ANTERIOR postural assessment with back and neck pain patients, what are the things you observe? You may choose more than one option.	28	26 had 100% agreement 1 had 75% agreement 1 had no agreement
8. LATERAL postural assessment with back and neck pain patients, what are the things you observe? You may choose more than one option.	18	6 had 100% agreement 6 had 50% agreement 1 had 33% agreement 1 had 20% agreement 4 had no agreement
9. Thinking only about patients with back or neck pain, which sort of pathologies do you come across commonly in your practice? Please note, the following are only some of the possible pathologies you may commonly come across. (Please tick as many as apply and use the ‘other’ box to add others).	41	26 had 100% agreement 1 had 83% agreement 1 had 66% agreement 1 had 62.5% agreement 6 had 50% agreement 2 had 33% agreement 3 had no agreement 1 response would not be rated
10. Is there anything else you would like to say about your use of postural assessment?	66	34 had 100% agreement 13 had 50% agreement 2 had 66% agreement 1 had 33% agreement 16 had no agreement
11. No free-text option	NA	NA

## Data Availability

The data that support the findings of this study are available from the corresponding author (R.D.) upon reasonable request. These data, due to confidentiality and ethical considerations, are not publicly available.

## Supplementary Materials

The following supporting information can be downloaded at: https://www.mdpi.com/article/10.3390/healthcare13243212/s1, File S1: Postural Assessment Survey Manual for Raters.
