# Supplementary material for: Postural Assessment: An Online Survey of Practicing Chiropractors in the UK
Source: Healthcare (Basel). 2025 Dec 8;13(24):3212. doi: 10.3390/healthcare13243212 (PMC12732692; doi:10.3390/healthcare13243212)
Supplement: Supplementary file 1 [file healthcare-13-03212-s001.zip › healthcare-3963777-supplementary.pdf]

Supplementary material for Duangkaew et al. Postural assessment: an online survey of practicing chiropractors in the UK

Percentage level of agreement between Raters for questions with free-text responses.

Survey question	Number of free-text responses	Percentage (%) agreement
Question 2	28	21 had 100% agreement<br>2 had 50% agreement<br>5 had no agreement
Question 4	17	16 had 100% agreement<br>1 had no agreement
Question 6	31	24 had 100% agreement<br>1 had 40% agreement<br>1 had 33% agreement<br>2 had 25% agreement<br>1 had 20% agreement<br>2 had zero agreement
Question 7	28	26 had 100% agreement<br>1 had 75% agreement<br>1 had no agreement
Question 8	18	6 had 100% agreement<br>6 had 50% agreement<br>1 had 33% agreement<br>1 had 20% agreement<br>4 had no agreement
Question 9	41	26 had 100% agreement<br>1 had 83% agreement<br>1 had 66% agreement<br>1 had 62.5% agreement<br>6 had 50% agreement<br>2 had 33% agreement<br>3 had no agreement<br>1 response would not be rated
**Question 10**	66	34 had 100% agreement<br>13 had 50% agreement<br>2 had 66% agreement<br>1 had 33% agreement

		16 had no agreement
**Total**	229	229

Postural Assessment Survey Manual for Raters

Instructions for raters
In this document you will find themes which have been developed to represent the free-text responses.

Each free-text question has its own set of themes.

Themes have been assigned a code. In some cases, the code it a letter, in some cases it is a number.

Please use the document titled Chiropractic Qualitative Responses and assign one or more codes to each response, for each question.

For some questions there are a lot of themes so to make it easier for you to identify these, themes have been grouped under headings according to the part of the body to which they relate. Within these headings they are alphabetized to further assist you.

If you are unable to assign a code to all or part of a response, please highlight or circle the bit you cannot assign a code to.

Explanations of unusual terms used by chiropractors have been included on the cover sheet where relevant to each question.

Themes/codes for Question 2

Question 2. As you answered 'always', 'frequently' or 'some of the time' to the previous question, please tick as many of the statements you agree with. I use postural assessment because:

A response may be assigned more than one code from the list below.

If you are unable to assign a code to all or part of a response, please highlight or circle the bit you cannot assign a code to.

**Theme**	**Code**
Is used because it informs the clinician or is for the benefit of the clinician	A
Is used because it informs the patient or is for the benefit of the patient	B
Is used because it is inherent to the job of being a chiropractor	C

Themes/codes for Question 4

Question 4: What methods do you use to carry out postural assessment? You may choose more than one option or tick as many as apply.

A response may be assigned more than one code from the list below.

If you are unable to assign a code to all or part of a response, please highlight or circle the bit you cannot assign a code to.

Theme	Code
App	A
Gonionmeter	B
Grid chart e.g. 'alignabod'	C
Mirror	D
Palpation	E
Photograph	F
Spinal Analyst Machine (SAM)	G
X-Ray	H

Themes/codes for Question 6

Question 6: If you perform POSTERIOR postural assessment with back- and neck pain patients, what are the things you observe?

You may choose more than one option.

A response may be assigned more than one code from the lists below.

If you are unable to assign a code to all or part of a response, please highlight or circle the bit you cannot assign a code to.

Theme	Code
**Face, head and neck**	
Ears; ear level	1
Head position	2
Neck position	3
Occipital ridge or occipital protuberance	4
Rotation of the cervical spine	5
**Upper limb**	
Position of forearms/hands	6
Scapula winging	7
Shoulder level	8
Shoulder position	9
Shoulder rotation	10
Shoulder tone	11
Shoulder torsion	12
Spine, torso and pelvis	
Global translations/CBP global subluxations	13

Kyphosis-lordosis; lateral spine shape	14
Lateral shift	15
Pelvic position	16
Pelvic tilt	17
Pelvic torsion	18
Rotation of the torso	19
Trunk Creases	20
Lower limb	
Foot arches	21
Lateral and medial malleoli	22
Leg length	23
Descriptions not specific to a particular body part	
Muscle tonicity	24
Relationship of one part of the body to another part	25
Rotation of a non-specified body part	26
Symmetry	27
Weight distribution	28

Themes/codes for Question 7

Question 7: If you perform ANTERIOR postural assessment with back- and neck pain patients, what are the things you observe?

You may choose more than one option.

A response may be assigned more than one code from the list below.

If you are unable to assign a code to all or part of a response, please highlight or circle the bit you cannot assign a code to.

**Theme**	**Code**
**Head, face and neck**	
Eye level	A
Head tilt	B
Rotation of cervical spine	C
TMJ/temporomandibular joint	D
**Upper limb**	
Forearm position	E
Hand position	F

Shoulder height	G
**Breast, chest and abdomen**	
Abdominal contours or size	H
Breast size	I
Pectoral muscle tone	J
Umbilicus	K
**Spine/torso**	
Rotation of thoracic spine	L
Rotation of torso	M
**Lower limb**	
Foot arches	N
Rotation of hip	O
Talus position	P
Toes	Q
**Responses not specific to one body part**	
Global subluxations	R
Muscle tone	S
Sway/weight/balance distribution	T
I don't use anterior assessment	U

Themes/codes for Question 8

Question 8. If you perform a LATERAL postural assessment with back- and neck pain patients, what are the things you observe?

You may choose more than one option.

A response may be assigned more than one code from the list below.

If you are unable to assign a code to all or part of a response, please highlight or circle the bit you cannot assign a code to.

**Theme**	**Code**
**Upper limb**	
Bulk of shoulder	A
**Breast/abdomen/pectoral**	
Abdomen size or shape	B
Breast size	C

Weak abdominals	D
Weak pectorals	E
**Upper limb**	
Acromioclavicular joint (AC)	F
**Spine**	
Dowager's hump	G
Spine shape	H
**Lower limb**	
Ankle/knee/hip relationship	I
Foot position	J
Knee flexion	K
**Items not specific to a body part**	
Plumb line Analysis	L
Rotation	M
Skin quality	N
weight distribution/ sway	O

Themes/codes for Question 9

Question 9. Thinking only about patients with back or neck pain, which sorts of pathologies do you come across commonly in your practice? Please note, the following are only some of the possible pathologies you may commonly come across. (Please tick as many as apply and use the 'other' box to add others).

A response may be assigned more than one code from the list below.

If you are unable to assign a code to all or part of a response, please highlight or circle the bit you cannot assign a code to.

Theme/code	Code
**Face/Head/Neck**	
Craniocervical dysfunction	1
Dizziness	2
Headaches	3
Sinusitis	4
TMJ pain	5
**Upper limb**	
Brachial plexus	6

Elbow dysfunction	7
Shoulder dysfunction or pain	8
Thoracic outlet syndrome	9
**Chest/Ribs**	
Costotransverse joint sprain	10
Costovertebral joint dysfunction/syndrome	11
Intercostal neuralgia	12
**Spine**	
Cauda equine syndrome	13
Disc derangement	14
Dural tube problem	15
Facet joint dysfunction	16
Low back pain	17
Spinal stenosis	18
Spondylolisthesis	19
Spondylosis	20
Upper cross syndrome	21
**Pelvis/groin**	
Pelvic instability	22
Pubic symphesis dysfunction or pain/SPD	23
Perineal pain	24
Sacroiliac/SI dysfunction or pain	25
**Lower limb**	
Ankle problems including ankle pain	26
Foot problems including foot pain	27
Hip problems including hip pain	28
Iliotibial band (ITB) pain	29
Knee problems including knee pain	30
Leg atrophy or weakness	31
Leg length difference	32
Lower leg trauma	33
Piriformis syndrome	34
Psoas spasm	35
Sciatica	36

**Items not specific to a part of the body**	
Amputation	37
Ankylosing spondylitis/AS	38
Arthritis; inflammatory arthropothies	39
Cancer	40
Enuresis	41
Ergonomic deformity or postural pain	42
Fatigue	43
Fractures	44
Hypermobility	45
Motor neurone syndrome	46
Myelopathy	47
Neuralgia	48
Occupational myalgia	49
Radiculopathy	50
Soft tissue problems/strains and sprains	51
Subluxation	52
Tension	53
Visceral pathologies	54
Weak core	55

Themes/codes for Question 10

Question 10. Is there anything else you would like to say about your use of postural assessment?

A response may be assigned more than one code from the list below.

If you are unable to assign a code to all or part of a response, please highlight or circle the bit you cannot assign a code to.

**Theme**	**Code**
Method of assessment	A
Specific items relating to the assessment of posture. For example, ‘shoulder height’, iliac crest’, ‘spine shape’.	B
Use and value of postural assessment (both positive and negative)/ rationale for assessment	C
Comment on the survey/study	D
